# The Organisation of Hospital Fire Brigades

**Published:** 1915-04-03

**Authors:** 


*To the Editor of* The Hospital.

### The Organisation of Hospital Fire Brigades.

*To the Editor of* The Hospital.

Sir,—We observe in your issue dated March 20 an article in which the statement is made that "Fire drills at hospitals are perfunctory affairs." We do not know on whose authority this statement is made, but we are able, from our own knowledge, to assert that there are very many hospitals where the nurses and all concerned take the keenest interest in fire drill, and show a very high degree of efficiency. We ourselves employ a staff of thoroughly competent men, ex-members of the London Fire Brigade, who, in addition to the knowledge of drill and the actual extinction of fires which they gained in that organisation, have had special training in our own workshops and under our technical staff in everything relating to the fire protection of buildings. These are the very best men who can possibly be selected as instructors, and we know that their services are very highly appreciated at many of the largest hospitals and other kindred institutions throughout the kingdom.

In regard to the National Fire Brigades Union, perhaps we may be allowed to point out that this body consists almost entirely of volunteer or amateur firemen. The chairman, who made the examination and granted the certificates referred to, is professionally a highly respected solicitor at Rugby. Your article says, further, that "the facts are very suggestive, and prove the necessity for periodical examinations by a central, outside, but recognised body." We think this remark is calculated to give the impression that the National Fire Brigades Union is an official body akin to a Government Department, or at least a society formed of professional firemen. It is a purely self-elected body, and, as we mentioned above, numbers very few professional firemen in its ranks. In our opinion, therefore, the result of handing over the control of fire inspections to such an organisation would be decidedly detrimental to the efficiency of many hospital fire brigades which now have the best training it is possible to obtain.

We quite realise that in writing the above we may be suspected of putting forward a case for our own commercial advantage, but we believe that any person who approaches the question with an open mind will agree on reflection that in the common interest, quite apart from such considerations, we are entitled to point out that the danger of loss of life by fire, which is always present to a greater or less extent in hospitals and other buildings where helpless people are lying, might easily be increased to a considerable extent if amateurs were to take over the control of the brigades formerly organised and instructed by professional firemen.—We are, Sir, your obedient servants,

Greenwich Road, S.E. Merryweather & Sons, Ltd.

